# Association between preoperative hematocrit and postoperative 30-day mortality in adult patients with tumor craniotomy

**DOI:** 10.3389/fneur.2023.1059401

**Published:** 2023-02-21

**Authors:** Yufei Liu, Lunzou Li, Haofei Hu, Jihu Yang, Xiejun Zhang, Lei Chen, Fanfan Chen, Shuyu Hao, Weiping Li, Guodong Huang

**Affiliations:** ^1^Shenzhen Key Laboratory of Neurosurgery, Department of Neurosurgery, The First Affiliated Hospital of Shenzhen University, Shenzhen Second People's Hospital, Shenzhen, China; ^2^Department of Neurosurgery, Beijing Tiantan Hospital, Capital Medical University, Beijing, China; ^3^Shenzhen University Health Science Center, Shenzhen, Guangdong, China; ^4^Department of Neurosurgery, Hechi People's Hospital, Hechi, Guangxi, China; ^5^Department of Nephrology, Shenzhen Second People's Hospital, The First Affiliated Hospital of Shenzhen University, Shenzhen, Guangdong, China

**Keywords:** hematocrit, brain tumor, craniotomy, steroid, mortality

## Abstract

**Background:**

The purpose of this research was to synthesize the American College of Surgeons National Surgical Quality Improvement Program database to investigate the link between preoperative hematocrit and postoperative 30-day mortality in patients with tumor craniotomy.

**Methods:**

A secondary retrospective analysis of electronic medical records of 18,642 patients with tumor craniotomy between 2012 and 2015 was performed. The principal exposure was preoperative hematocrit. The outcome measure was postoperative 30-day mortality. We used the binary logistic regression model to explore the link between them and conducted a generalized additive model and smooth curve fitting to investigate the link and its explicit curve shape. We conducted sensitivity analyses by converting a continuous HCT into a categorical variable and calculated an E-value.

**Results:**

A total of 18,202 patients (47.37% male participants) were included in our analysis. The postoperative 30-day mortality was 2.5% (455/18,202). After adjusting for covariates, we found that preoperative hematocrit was positively associated with postoperative 30-day mortality (OR = 0.945, 95% CI: 0.928, 0.963). A non-linear relationship was also discovered between them, with an inflection point at a hematocrit of 41.6. The effect sizes (OR) on the left and right sides of the inflection point were 0.918 (0.897, 0.939) and 1.045 (0.993, 1.099), respectively. The sensitivity analysis proved that our findings were robust. The subgroup analysis demonstrated that a weaker association between preoperative hematocrit and postoperative 30-day mortality was found for patients who did not use steroids for chronic conditions (OR = 0.963, 95% CI: 0.941–0.986), and a stronger association was discovered in participants who used steroids (OR = 0.914, 95% CI: 0.883–0.946). In addition, there were 3,841 (21.1%) cases in the anemic group (anemia is defined as a hematocrit (HCT) <36% in female participants and <39% in male participants). In the fully adjusted model, compared with the non-anemic group, patients in the anemic group had a 57.6% increased risk of postoperative 30-day mortality (OR = 1.576; 95% CI: 1.266, 1.961).

**Conclusion:**

This study confirms that a positive and nonlinear association exists between preoperative hematocrit and postoperative 30-day mortality in adult patients undergoing tumor craniotomy. Preoperative hematocrit was significantly associated with postoperative 30-day mortality when the preoperative hematocrit was <41.6.

## Background

Craniotomies are a common neurological practice for intracranial tumor resections. However, craniotomies for brain tumors carry significant risks of mortality and morbidity (1). The postoperative 30-day mortality (also called 30-day postoperative mortality) provides an effective and objective evaluation of access to and safe operation and anesthesia (2). Postoperative 30-day mortality ranged from 0.95 to 8.62% based on a study of English patients with a brain tumor who underwent a craniotomy during 2008–2010 (3). Hankinson et al. (4) reported that the postoperative 30-day mortality of diagnostic neurosurgery for a primary childhood brain tumor was between 1.16 and 1.72%, and the mortality of a U.S. pediatric population was roughly in line with contemporary data from European populations (5). Anemia, as an indicator of many diseases, is defined as a hematocrit (HCT) <36% in female participants and <39% in male participants (6, 7). Accumulating studies have shown that preoperative anemia is associated with an increased risk of mortality or postoperative complications in patients experiencing cardiac surgery (8) and non-cardiac surgery (9–12). Increasing evidence indicates that a low preoperative HCT is an indicator of a poor prognosis in patients undergoing non-cardiac surgery (4, 13–16). Recent research reported that a lower preoperative HCT was correlated with a neurocognitive decline in patients undergoing cardiac surgery on cardiopulmonary bypass (17).

Few clinical studies have explored the association between preoperative low HCT and prognosis in patients with craniocerebral surgery, and their findings are controversial. A lower preoperative HCT was more likely to be associated with medical complications in patients who underwent ventral skull base surgery (18). A lower preoperative HCT was also a predictor of medical complications in patients undergoing transnasal microscopic pituitary tumor excision (19). However, another study showed that preoperative anemia was not associated with an overall increased risk for a poor prognosis in patients with elective craniotomy (20). In addition, red blood cell (RBC) transfusions increase HCT levels (21). Elevated HCT concentrations were associated with an increased risk of venous thrombosis and postoperative 30-day mortality following major surgery (22). RBC transfusion during cranial surgery was associated with postoperative complications, 30-day reoperation, prolonged length of hospitalization, and postoperative 30-day mortality (23). Hence, it is important to identify the potential associations between preoperative HCT levels and the outcomes of patients with brain tumor undergoing craniotomy.

To date, no research has investigated the possibility of a nonlinear relationship or conducted subgroup analyses between preoperative HCT and 30-day postoperative mortality. The link between them in patients with tumor craniotomy remains to be explored. Thus, this study aimed to examine their relationship from data from a cross-sectional study based on the American College of Surgeons National Surgical Quality Improvement Program (ACS NSQIP) database. This study could serve as a resource for clinicians regarding the role of preoperative HCT in assessing short-term postoperative outcomes of patients with tumor craniotomy.

## Participants and methods

### Study design

We utilized data from the ACS NSQIP database (2012–2015) for this cross-sectional study. In our study, the independent and dependent variables were preoperative HCT and postoperative 30-day mortality, respectively.

### Dataset source

The raw data were from the ACS NSQIP database (https://www.facs.org/quality-programs/acs-nsqip). Zhang et al. (24), the authors of “Sepsis and septic shock after craniotomy: Predicting a significant patient safety and quality outcome measure” (10.1371/journal.pone.0235273) (24), originally uploaded the ACS NSQIP database (2012–2015). The original study was published as an open access article under the Creative Commons Attribution License, which permits unrestricted distribution, use, and reproduction. Therefore, our study could use the uploaded database for secondary analysis without violating the rights of the authors.

### Participants

**Figure 1:**
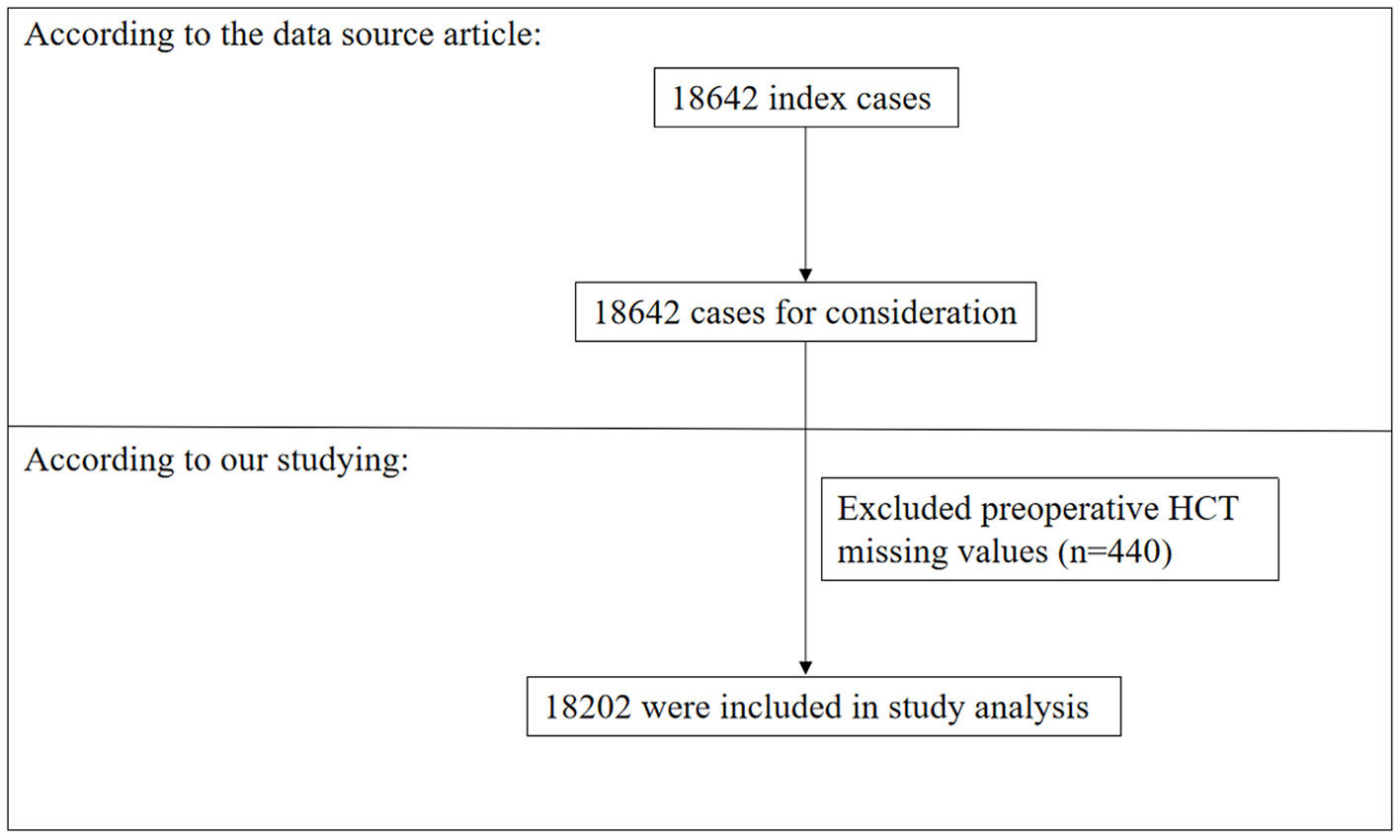
Flowchart of the study participants.

The original research included 18,642 adult participants with brain tumors. A total of 18,202 participants were included in our analysis after excluding participants with missing values for preoperative HCT (*n* = 440) (see the flowchart for details in Figure 1). Because our research was a secondary analysis of the historical database and the original personal information was not identified, consent forms were not needed.

### Variables

Preoperative HCT (%) was recorded as a continuous variable in the originally uploaded data (24). Data collected for HCT were analyzed by the Current Procedural Terminology code.

### Thirty-day postoperative mortality

Originally, the authors defined postoperative 30-day mortality as mortality within the first 30 days after surgery (24). NSQIP tracked mortality for the first 30 postoperative days.

### Covariates

Our choice of covariates was based on previous literature reports and our clinical experience. Weight (kg) divided by height (m) squared (kg/m^2^) was used to calculate the body mass index (BMI). Standard conditions were followed when collecting and treating the original data. The continuous variables in this study were as follows: BMI and indicators of preoperative blood tests [serum sodium (Na), blood urea nitrogen (BUN), creatine (Cr), white blood cell (WBC) count, and platelet (PLT) count]. The categorical variables were as follows: race (white, Asian, African American, or unknown race), sex (male or female), age range (18–40, 41–60, 60–80, or > 80 years old), a history of diabetes [no, yes (insulin-dependent), or yes (non-insulin-dependent)], smoking status (no or yes), severe chronic obstructive pulmonary disease (COPD) (no or yes), preoperative transfusions (no or yes), congestive heart failure (CHF) (no or yes), hypertension (no or yes), dialysis (no and yes), disseminated cancer (no or yes), steroid use for a chronic condition (no or yes), preoperative systemic sepsis (no, systemic inflammatory response syndrome (SIRS), or septic shock), and bleeding disorders (no or yes). The authors provided more details in the original research (24). In addition, the continuous variable WBC count was converted into dichotomous variables according to low (WBC count ≤ 10 × 10^9^/L) or high risk (WBC count > 10 × 10^9^/L) (25) for subgroup analyses.

### Statistical analysis

The number of cases with missing values for BMI, Na, BUN, Cr, WBC, and PLT were 719 (3.95%), 471 (2.59%), 1,176 (6.46%), 395 (2.17%), 164 (0.9%), and 159 (0.87%), respectively. Median or mean values were used instead of missing values for statistical analysis.

Included participants were stratified by quartiles of preoperative HCT. We reported the mean ± standard deviation (SD) (normally distributed variables) or median (interquartile range) (nonnormally distributed variables) for continuous variables; we extracted percentages and frequencies for categorical outcomes. We used the χ^2^ tests (for categorical variables), the Kruskal–Wallis *H*-tests (for nonnormally distributed data), or the one-way ANOVAs (for normally distributed variables) to test for significant differences among the different HCT groups. To explore the relationship between preoperative HCT and postoperative 30-day mortality, distinctive univariate, and multivariate binary logistic regression models were established complying with the Strengthening the Reporting of Observational Studies in Epidemiology (STROBE) statement guidelines. The models included a non-adjusted model (not adjusted for any covariates), a minimally adjusted model (only adjusted for age range, race, and sex), and a fully adjusted model (adjusted for BMI, sex, race, age range, diabetes, smoking status, CHF, COPD, hypertension, dialysis, disseminated cancer, bleeding disorders, steroid use, preoperative transfusions, preoperative systemic sepsis, serum Na, BUN, Cr, WBC count, and PLT count) for the covariates presented in Table 1. We recorded 95% confidence intervals and effect sizes and adjusted the effect sizes when covariates were added to the model and the OR changed by 10% or more (26). Anemia is defined as a hematocrit (HCT) <36% in female participants and <39% in male participants (6, 7). Included participants were stratified according to the definition of anemia. We constructed three multivariate binary logistic regression models to examine the link between preoperative anemia and postoperative 30-day mortality.

**Table 1:** Baseline demographic characteristics of the participants.

**HCT (quartile) %**	**Q1 (10.600–37.500)**	**Q2 (37.600–40.600)**	**Q3 (40.700–43.400)**	**Q4 (43.500–60.000)**	***P*-value**
*N*	4,532	4,529	4,503	4,638	
BMI (Mean ± SD)	28.06 ± 7.07	28.54 ± 6.73	28.94 ± 6.37	29.30 ± 6.19	<0.001
Na (Mean ± SD)	138.43 ± 3.54	138.73 ± 3.1	138.68 ± 3.04	138.66 ± 3.02	<0.001
BUN (Median Q1-Q3)	16.00 (12.00–21.00)	16.00 (12.00–20.00)	16.00 (13.00–20.00)	16.00 (13.00–20.00)	<0.001
Cr (Median Q1-Q3)	0.76 (0.62–0.92)	0.79 (0.67–0.90)	0.80 (0.70–0.93)	0.89 (0.78–1.00)	<0.001
WBC (Median Q1-Q3)	8.30 (6.10–11.20)	8.200 (6.20–11.10)	8.40 (6.50-11.50)	9.10 (6.90-12.60)	<0.001
PLT (Median Q1-Q3)	237.00 (186.00–295.00)	238.00 (197.00–287.00)	239.00 (198.00–284.00)	234.00 (195.00–275.00)	<0.001
Sex N (%)					<0.001
Male	1,414 (31.200%)	1,496 (33.032%)	2,244 (49.833%)	3,468 (74.774%)	
Female	3,118 (68.800%)	3,033 (66.968%)	2,259 (50.167%)	1,170 (25.226%)	
Race N (%)					<0.001
White	3,087 (68.116%)	3,261 (72.003%)	3,230 (71.730%)	3,362 (72.488%)	
Asian	135 (2.979%)	132 (2.915%)	141 (3.131%)	121 (2.609%)	
African American	535 (11.805%)	287 (6.337%)	209 (4.641%)	197 (4.248%)	
Unknown race	775 (17.101%)	849 (18.746%)	923 (20.497%)	958 (20.655%)	
Age ranges N (%)					<0.001
18–40	601 (13.261%)	691 (15.257%)	753 (16.722%)	901 (19.426%)	
41–60	1,757 (38.769%)	1,932 (42.658%)	1,906 (42.327%)	1,959 (42.238%)	
61–80	1,943 (42.873%)	1,758 (38.817%)	1,722 (38.241%)	1,675 (36.115%)	
> 81	231 (5.097%)	148 (3.268%)	122 (2.709%)	103 (2.221%)	
Diabetes N (%)					<0.001
No	3,811 (84.091%)	3,991 (88.121%)	4,041 (89.740%)	4,217 (90.923%)	
Yes (Non-insulin)	428 (9.444%)	358 (7.905%)	297 (6.596%)	268 (5.778%)	
Yes (Insulin)	293 (6.465%)	180 (3.974%)	165 (3.664%)	153 (3.299%)	
Smoking status N (%)					<0.001
No	3,676 (81.112%)	3,716 (82.049%)	3,680 (81.723%)	3,604 (77.706%)	
Yes	856 (18.888%)	813 (17.951%)	823 (18.277%)	1,034 (22.294%)	
Severe COPD N (%)					<0.001
No	4,223 (93.182%)	4,347 (95.981%)	4,333 (96.225%)	4,477 (96.529%)	
Yes	309 (6.818%)	182 (4.019%)	170 (3.775%)	161 (3.471%)	
Congestive heart failure N (%)					0.005
No	4,507 (99.448%)	4,514 (99.669%)	4,490 (99.711%)	4,632 (99.871%)	
Yes	25 (0.552%)	15 (0.331%)	13 (0.289%)	6 (0.129%)	
Hypertension N (%)					<0.001
No	2,538 (56.002%)	2,804 (61.912%)	2,885 (64.068%)	3,008 (64.856%)	
Yes	1,994 (43.998%)	1,725 (38.088%)	1,618 (35.932%)	1,630 (35.144%)	
Dialysis					<0.001
No	4,483 (98.919%)	4,525 (99.912%)	4,501 (99.956%)	4,637 (99.978%)	
Yes	49 (1.081%)	4 (0.088%)	2 (0.044%)	1 (0.022%)	
Disseminated cancer N (%)					<0.001
No	2,886 (63.680%)	3,600 (79.488%)	3,754 (83.367%)	3,988 (85.985%)	
Yes	1,646 (36.320%)	929 (20.512%)	749 (16.633%)	650 (14.015%)	
Steroid use for chronic condition N (%)					<0.001
No	3,744 (82.613%)	3,886 (85.803%)	3,867 (85.876%)	3,962 (85.425%)	
Yes	788 (17.387%)	643 (14.197%)	636 (14.124%)	676 (14.575%)	
Bleeding disorders					<0.001
No	4,358 (96.161%)	4,459 (98.454%)	4,435 (98.490%)	4,577 (98.685%)	
Yes	174 (3.839%)	70 (1.546%)	68 (1.510%)	61 (1.315%)	
Preoperation transfusions N (%)					<0.001
No	4,481 (98.875%)	4,524 (99.890%)	4,496 (99.845%)	4,638 (100.000%)	
Yes	51 (1.125%)	5 (0.110%)	7 (0.155%)	0 (0.000%)	
Preoperation systemic sepsis N (%)					<0.001
No	4,272 (94.263%)	4,371 (96.511%)	4,373 (97.113%)	4,515 (97.348%)	
SIRS	255 (5.627%)	157 (3.467%)	126 (2.798%)	122 (2.630%)	
Septic Shock	5 (0.110%)	1 (0.022%)	4 (0.089%)	1 (0.022%)	
30-day mortality events, N (%)					<0.001
No	4,352 (96.028%)	4,425 (97.704%)	4,423 (98.223%)	4,547 (98.038%)	
Yes	180 (3.972%)	104 (2.296%)	80 (1.777%)	91 (1.962%)	

We conducted a generalized additive model (GAM) and smooth curve fitting to address the specific link between the non-linear relationship between preoperative HCT and 30-day postoperative mortality. If non-linearity was detected, the inflection point was calculated using a recursive algorithm, and a piecewise binary logistic regression model was subsequently constructed with one piece on each side of the inflection point. We employed the log-likelihood ratio test to determine the most suitable model for elaborating the link between them. We followed the methods of Liu et al. (27).

We executed subgroup analyses using a stratified binary logistic regression model for many subgroups, including sex, race, age range, smoking status, steroid use for chronic conditions, diabetes, severe COPD, hypertension, disseminated cancer, WBC count, and emergency cases. First, we converted the WBC count (continuous variable) to a categorical variable according to the abovementioned clinical threshold (≤ 10 × 10^9^/L, >10 × 10^9^/L) (25). Second, we adjusted each stratification for all factors (sex, race, BMI, age range, diabetes, smoking status, COPD, CHF, hypertension, dialysis, steroid use, bleeding disorders, preoperative transfusions, disseminated cancer, preoperative systemic sepsis, serum Na, BUN, Cr, WBC count, and PLT count), except for the stratification factor. We finally tested for interactions by employing the likelihood ratio test for models with and without interaction terms (28, 29). We followed the methods of Liu et al. (27).

We performed sensitivity analyses to verify the robustness of our findings. We converted HCT into a categorical variable according to its quartiles and then calculated the *P*-value for each trend to verify the results of using HCT as a continuous variable and to examine the possibility of non-linearity. We subsequently investigated the potential for unknown confounders on the link between HCT and 30-day postoperative mortality by calculating E-values (30). We deleted the data of the participants with the missing values, and then re-analyzed the data of 16,245 participants. All results were reported in compliance with the STROBE statement guidelines. We followed the methods of Liu et al. (27).

The statistical analyses were performed using the statistical software EmpowerStats (http://www.empowerstats.com, X&Y Solutions, Inc., Boston, MA) and the R package (http://www.R-project.org, The R Foundation). For all the tests, a two-sided *p* < 0.05 was considered statistically significant.

## Results

### Baseline characteristics of the participants

The demographic and clinical characteristics of the study participants are provided in Table 1. A total of 18,202 cases (47.37% male) were included in our analysis. The age distribution proportions were 16.18% (18–40), 41.5% (41–60), 39.0% (61–80), and 3.32% (>81). The mean preoperative HCT value was 40.32 ± 4.81%. The postoperative 30-day mortality rate of the included patients was 2.5% (455/18,202). We assigned the included cases into subgroups using HCT quartiles: Q1 (10.600–37.500), Q2 (37.600–40.600), Q3 (40.700–43.400), and Q4 (43.500–60.000). Compared with those of the included cases with a lower HCT (10.600–37.500), the highest HCT (43.500–60.000) was significantly positively correlated with sex, age range, BMI, diabetes, race, smoking status, severe COPD, CHF, hypertension, dialysis, steroid use, disseminated cancer, bleeding disorders, preoperative transfusions, preoperative systemic sepsis, serum Na, BUN, Cr, WBC count, and PLT count (all *P*-values of < 0.05).

### The results of univariate analyses using a binary logistic regression model

The univariate analysis was performed, indicating that in participants who were female, 41–60 years old, 61–80 years old, >81 years old, and with diabetes (noninsulin-dependent and insulin-dependent), severe COPD, CHF, hypertension, dialysis, disseminated cancer, steroid use, bleeding disorders, preoperative transfusions, preoperative SIRS, preoperative septic shock, levels of Na, BUN, WBC, PLT, and HCT were positively associated with postoperative 30-day mortality. In contrast, patients who were Asian, African American, of unknown race, and those who smoked were not associated with postoperative 30-day mortality (Supplementary Table 1).

### The results of multivariate analyses using the binary logistic regression model

We established three binary logistic regression models to explore the link between preoperative HCT and postoperative 30-day mortality. We found a significant association between them in three binary logistic regression models (Table 2). In the unadjusted model, we found that a decrease of 1 unit of preoperative HCT was related to a 7.6% increase in postoperative 30-day mortality [OR = 0.924; 95% CI: (0.908, 0.939)], which was statistically significant. In the minimally adjusted model, when we only adjusted for sex, age range, and race, each decreased preoperative HCT unit may result in elevated postoperative 30-day mortality by 7.9% [OR = 0.921; 95% CI: (0.905, 0.937)]. The findings on the link between HCT and 30-day postoperative mortality obtained from the model were statistically significant. In the fully adjusted model, a decrease of 1 unit of preoperative HCT was accompanied by a 5.5% increase in 30-day postoperative mortality[(OR = 0.945; 95% CI: 0.928, 0.963)]. In addition, there were 3,841 (21.1%) cases in the anemic group (anemia is defined as a hematocrit (HCT) <36% in female participants and <39% in male participants). We found a significant association between preoperative anemia and postoperative 30-day mortality in three binary logistic regression models. In the nonadjusted model, compared with the non-anemic group, patients in the anemic group had a 149.4% increased risk of postoperative 30-day mortality (OR = 2.494; 95% CI: 2.060, 3.021). In the fully adjusted model, compared with the non-anemic group, patients in the anemic group had a 57.6% increased risk of postoperative 30-day mortality (OR = 1.576; 95% CI: 1.266, 1.961). The distribution of CIs suggested that the relationship between them obtained by the model was reliable (see Table 2 for details).

**Table 2:** Relationship between preoperative HCT and 30-day postoperative mortality in the three binary logistic regression models.

**Exposure**	**Model 1 (OR, 95% CI, P)**	**Model 2 (OR, 95% CI, P)**	**Model 3 (OR, 95% CI, P)**
**HCT**	0.924 (0.908, 0.939) <0.001	0.921 (0.905, 0.937) <0.001	0.945 (0.928, 0.963) <0.001
**HCT (quartile)**			
**Q1**	Ref	Ref	Ref
**Q2**	0.568 (0.445, 0.726) <0.001	0.599 (0.468, 0.768) <0.001	0.753 (0.582, 0.974) 0.031
**Q3**	0.437 (0.335, 0.571) <0.001	0.429 (0.327, 0.564) <0.001	0.567 (0.427, 0.753) <0.001
**Q4**	0.484 (0.375, 0.625) <0.001	0.442 (0.338, 0.579) <0.001	0.608 (0.457, 0.808) <0.001
**P for trend**	<0.001	<0.001	<0.001
**Anemia**			
**No**	Ref	Ref	Ref
**Yes**	2.494 (2.060, 3.021) <0.001	2.167 (1.781, 2.636) <0.001	1.576 (1.266, 1.961) <0.001

Model 1 (nonadjusted model): not adjusted for any covariates. Model 2 (minimally adjusted model): adjusted for sex, race, and age ranges. Model 3 (fully adjusted model): adjusted for sex, race, BMI, age range, CHF, diabetes, smoking status, severe COPD, dialysis, hypertension, steroid use for chronic conditions, disseminated cancer, bleeding disorders, preoperative transfusions, preoperative systemic sepsis, serum Na, BUN, Cr, WBC, and PLT. Ref, reference; OR, odds ratios; CI, confidence interval. Anemia is defined as a hematocrit (HCT) <36% in female participants and <39% in male participants.

### Sensitivity analyses

We conducted sensitivity analyses to test the robustness of our results. We first converted a continuous HCT into a categorical variable (divided into groups according to quartiles) and then changed the previous HCT variable in the model to the categorical-transformed HCT. After HCT was transformed into a categorical variable, the trend of the effect sizes in different groups was equidistant, and the P for the trend was consistent with the result when HCT was a continuous variable. In addition, we used a GAM to insert the continuity covariate as curves into the equation. To assess the sensitivity to unmeasured confounders, we also calculated an E-value of 1.31. It was greater than the relative risk of unmeasured confounders influencing the link between HCT and postoperative 30-day mortality, indicating that unknown or unmeasured confounders had little effect on the link between them. In addition, we deleted the data of the participants with the missing values and then re-analyzed the data of 16,245 participants. The postoperative 30-day mortality was 2.54% (412/16,245). We observed a similar significant association between HCT and postoperative 30-day mortality in three binary logistic regression models (see Supplementary Table 2 for details).

### The nonlinearity addressed by the generalized additive model

**Figure 2:**
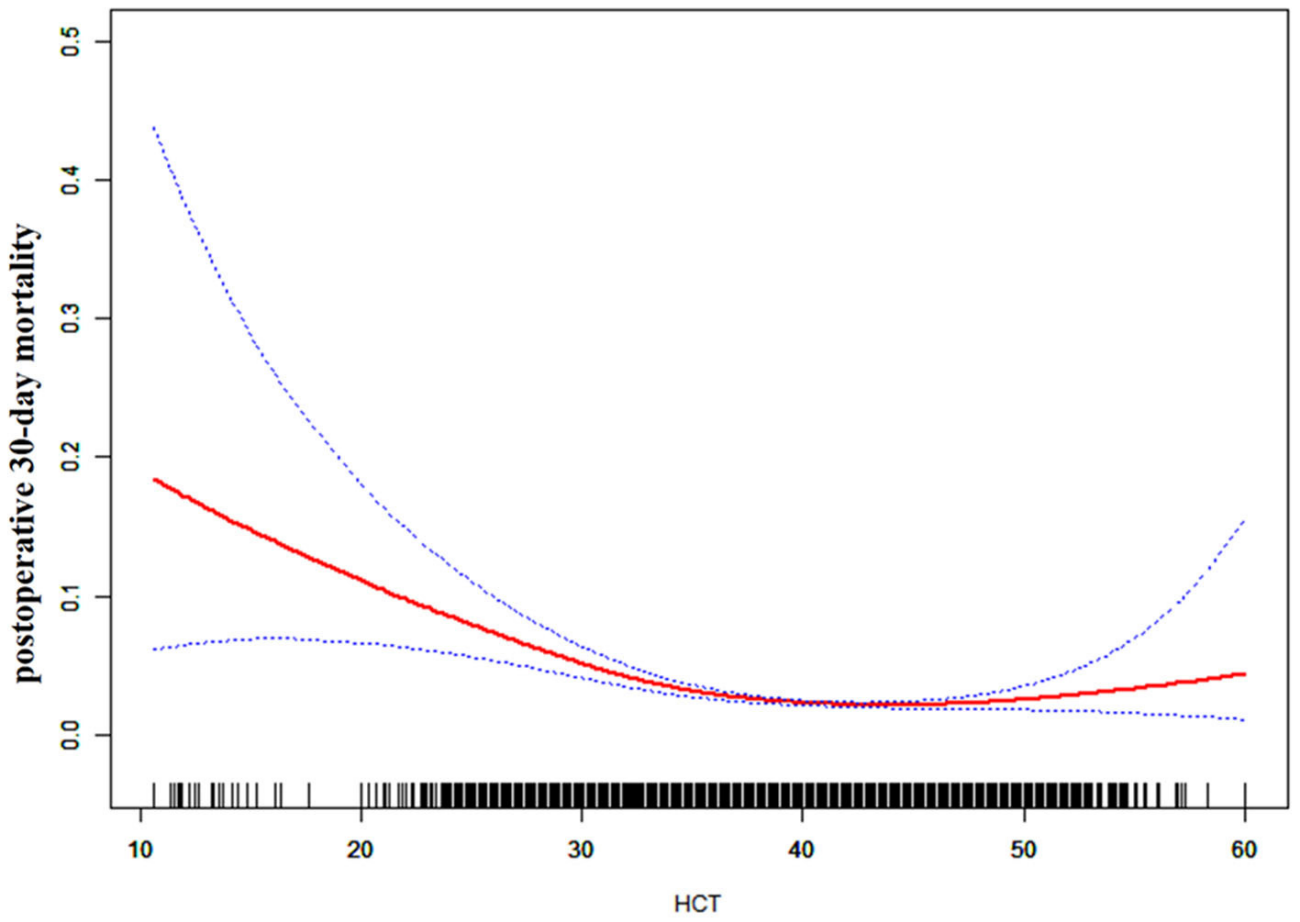
The non-linear relationship between preoperative HCT and the risk of 30-day postoperative mortality (blue line: confidence interval, red line: postoperative 30-day mortality).

We found that the relationship between preoperative HCT and postoperative 30-day mortality was non-linear through GAM and smooth curve fitting (as shown in Figure 2). Hence, we fit the data to a piecewise binary logistic regression model that allowed two different slopes. Data were also fitted by a standard binary logistic regression model based on the sensitivity analysis, and the best-fit model was chosen through the log-likelihood ratio test (as shown in Table 3). The P for the log-likelihood ratio test was <0.05 in our analysis. A piecewise model was used to fit the association between HCT and postoperative 30-day mortality. With a recursive algorithm, we obtained an inflection point of 41.6 for the first time and then computed the effect sizes and CIs to the right and left of the inflection point with the piecewise binary logistic regression model. The effect size (OR) was 1.045, and the 95% CI was from 0.993 to 1.099 on the right side of the inflection point. The OR was 0.918, and the 95% CI was from 0.897 to 0.939 on the left side of the inflection point.

**Table 3:** The results of the two piecewise linear regression models.

	**Postoperative 30-day mortality (OR, 95% CI, P-value)**
**Fitting the model by standard linear regression**	0.945 (0.928, 0.963) <0.001
**Fitting the model by two-piecewise linear regression**	
**Infection point of HCT**	41.6
**≥41.6**	0.918 (0.897, 0.939) <0.001
**>41.6**	1.045 (0.993, 1.099) 0.090
**P-value for log likelihood ratio test**	<0.001

This model adjusted for sex, race, BMI, age range, CHF, diabetes, smoking status, severe COPD, dialysis, hypertension, steroid use for chronic conditions, disseminated cancer, bleeding disorders, preoperative transfusions, preoperative systemic sepsis, serum Na, BUN, Cr, WBC, and PLT. OR, odds ratios; CI, confidence interval.

### Subgroup analyses

We conducted subgroup analyses in view of other factors that might influence the link between preoperative HCT and postoperative 30-day mortality. We employed stratification variables (including age range, sex, race, diabetes, smoking status, severe COPD, CHF, hypertension, steroid use, disseminated cancer, and WBC count) to detect the trend of effect sizes. No significant differences were found in the relationship in different sex, age range, diabetes, race, smoking status, severe COPD, CHF, hypertension, disseminated cancer, or WBC groups (P for interaction > 0.05); only steroid use for chronic conditions modified the link between preoperative HCT and postoperative 30-day mortality (P for interaction <0.05). A weaker association was found for patients who did not use steroids for chronic conditions (OR = 0.963, 95% CI: 0.941–0.986), and a stronger association was discovered in participants who did use steroids (OR = 0.914, 95% CI: 0.883–0.946) (see Table 4 for details).

**Table 4:** Effect size of preoperative HCT on 30-day postoperative mortality in prespecified and exploratory subgroups.

**Characteristic**	**No. of participants**	**OR (95% CI)**	**P for interaction**
Sex			0.254
Male	8,622	0.939 (0.918, 0.961)	
Female	9580	0.960 (0.931, 0.991)	
Age ranges			0.097
18-40	2,946	0.977 (0.871, 1.095)	
41-60	7,554	0.933 (0.902, 0.965)	
61-80	7,098	0.940 (0.917, 0.964)	
> 81	604	1.021 (0.955, 1.091)	
Race			0.069
White	12,940	0.932 (0.912, 0.953)	
Asian	529	1.034 (0.867, 1.233)	
African American	1,228	1.003 (0.928, 1.083)	
Unknown race	3,505	0.977 (0.935, 1.021)	
Diabetes			0.608
No	16,060	0.947 (0.927, 0.968)	
Yes (Noninsulin)	1,351	0.920 (0.872, 0.971)	
Yes (Insulin)	791	0.948 (0.890, 1.009)	
Smoking status			0.801
No	14,676	0.945 (0.925, 0.966)	
Yes	3,526	0.940 (0.904, 0.977)	
Severe COPD			0.591
No	17,380	0.950 (0.931, 0.969)	
Yes	822	0.934 (0.884, 0.988)	
CHF			0.509
No	18,143	0.946 (0.928, 0.964)	
Yes	59	0.910 (0.811, 1.021)	
Hypertension			0.331
No	11,235	0.936 (0.909, 0.964)	
Yes	6,967	0.954 (0.931, 0.978)	
Dialysis			0.482
No	18,146	0.942 (0.925, 0.960)	
Yes	56	1.003 (0.845, 1.190)	
Disseminated cancer			0.109
No	14,228	0.962 (0.936, 0.988)	
Yes	3,974	0.932 (0.907, 0.958)	
Steroid use for a chronic condition			0.013
No	15,459	0.963 (0.941, 0.986)	
Yes	2,743	0.914 (0.883, 0.946)	
WBC			0.475
WBC ≤ 10	11,621	0.939 (0.913, 0.966)	
WBC >10	6,581	0.952 (0.928, 0.977)	

## Discussion

We utilized the ACS NSQIP database from 2012 to 2015 to investigate whether there was a link between preoperative HCT and postoperative 30-day mortality in this study. To the best of our knowledge, the present data analysis is the first to observe a non-linear link between them. The authors used a two-piecewise linear regression model to clarify a non-linear link between them. The inflection point was 41.6 after adjusting for confounders. When the preoperative HCT was below 41.6%, a one-unit decrease in preoperative HCT was related to an 8.2% increase in the postoperative 30-day mortality [OR = 0.918, 95% CI (0.897, 0.939)], which may indicate that increasing the preoperative HCT level could significantly reduce postoperative 30-day mortality when the preoperative HCT level is below the inflection point. RBC transfusions increase HCT levels (21). However, elevated HCT concentrations were associated with an increased risk of venous thrombosis and postoperative 30-day mortality following major surgery (22), and associated with postoperative complications, 30-day reoperation, prolonged length of hospitalization, and postoperative 30-day mortality during cranial surgery (23), which should be of particular concern to doctor in RBC transfusion to raise HCT. However, we were able to establish an association between preoperative HCT and postoperative 30-day mortality, controlling for preoperative transfusions and other factors. These results suggested that, except for rapid changes in acute blood transfusion and blood loss, chronic changes in HCT were still associated with postoperative 30-day mortality. When preoperative HCT > 41.6%, no significant link was observed between them (*P*-value > 0.05). We speculated that patients with lower preoperative HCT might have lower postoperative HCT under the influence of blood loss during surgery, increasing the risk of 30-day postoperative mortality. However, patients with a higher preoperative HCT may tolerate a certain degree of blood loss during surgery, resulting in no significant impact on postoperative 30-day mortality.

The authors found that steroid use for chronic conditions could serve as a potential effect modifier to modify the link between preoperative HCT and postoperative 30-day mortality in subgroup analysis. Stronger associations were found in patients with steroid use for chronic conditions. In comparison, significantly weaker links were detected in patients without steroid use for chronic conditions. To date, clinical studies on the link between preoperative steroid use and outcomes in patients with intracranial tumors are scarce, and their findings are debatable. Medical management for intracranial tumors usually includes the administration of corticosteroids to control peritumoral edema and treat its associated neurologic deficits (31, 32). Unfortunately, corticosteroids are associated with numerous and long-term side adverse effects, constituting a significant challenge in patients who need long-term corticosteroid use (31, 33). Lieber et al. (34) maintained that preoperative corticosteroid use and chemotherapy were independent predictors of organ-space surgical-site infections in patients undergoing cranial neurosurgery (34). The results of our study indicated that chronic preoperative steroid therapy modifies the link between HCT and postoperative 30-day mortality in U.S. adult patients with tumor craniotomy. The aforementioned results indicate that chronic preoperative steroid therapy affects the postoperative short-term outcome in patients with tumor craniotomy.

Our study has some strengths as follows: (1) It is the large sample size that allows such analysis. Most covariates have complete information, with few missing data. (2) To the best of our knowledge, this is the first study to observe the association between preoperative serum HCT and postoperative 30-day mortality in patients with tumor craniotomy. (3) We found a nonlinear relationship between them; thus, our study has greater clinical significance, which previous studies have not investigated. (4) We employed interaction analysis and subgroup analysis. (5) We used sensitivity analyses to test the robustness of our findings.

Our research has the following shortcomings: (1) this was a retrospective study on a national database; (2) because our research was a secondary analysis of previously published data, we could not exclude some residual and/or unmeasured confounding factors that could interfere with the estimated link (e.g., characteristics of benign and malignant tumors, different types of brain tumors, pharmacological treatments, and socioeconomic factors). However, we computed the E-value to quantify the potential influence of unmeasured confounders and concluded that they were unlikely to explain the findings; (3) we could also not investigate the link between preoperative serum HCT and long-term prognosis; and (4) the absence of hemoglobin (Hb) and mean corpuscular volume (MCV) was one of the limitations due to the nature of the secondary analysis. This research was based on collected data from a heterogeneous and large sample size. Therefore, the link and results postulated remain highly plausible.

## Conclusion

This study confirmed a positive and non-linear link between preoperative HCT and postoperative 30-day mortality in patients with tumor craniotomy and a threshold effect between them. A significant positive association with postoperative 30-day mortality was discovered when the preoperative HCT was lower than 41.6. These findings are expected to provide suitable guidance for clinicians (especially neurosurgeons) for preoperative HCT. Increasing the preoperative HCT level can significantly reduce postoperative 30-day mortality when the preoperative HCT level is below the inflection point. Consequently, abnormal preoperative HCT signals may assist clinicians in identifying high-risk populations for 30-day postoperative mortality in this particular population, which will allow clinicians to plan and initiate appropriate management strategies. However, the problems caused by preoperative RBC transfusion to raise HCT should also be a concern.

## Data availability statement

The original contributions presented in the study are included in the article/Supplementary material, further inquiries can be directed to the corresponding author.

## Ethics statement

The studies involving human participants were reviewed and approved by the Clinical Research Ethics Committee of Shenzhen Second People's Hospital. Written informed consent for participation was not required for this study in accordance with the national legislation and the institutional requirements.

## Author contributions

YL and HH analyzed the data, performed the statistical analysis, and contributed to the interpretation of the data. YL, LL, and HH prepared the original manuscript. FC, SH, WL, and GH reviewed and edited the final version of the manuscript. XZ, JY, and LC confirmed the authenticity of all of the raw data. All authors have read and approved the final manuscript.
