# Supplementary material for: Association between preoperative hematocrit and postoperative 30-day mortality in adult patients with tumor craniotomy
Source: Front Neurol. 2023 Feb 21;14:1059401. doi: 10.3389/fneur.2023.1059401 (PMC9990837; doi:10.3389/fneur.2023.1059401)
Supplement: Supplementary file 1 [file Table_1.DOCX]

**Supplementary table 1** **The results of the univariate analysis**

|  | **Statistics** | **OR** | **95% CI** | **P value** |
| --- | --- | --- | --- | --- |
| **Sex, N (%)** |  |  |  |  |
| **Male** | 8622 (47.37%) | Ref. |  |  |
| **Female** | 9580 (52.63%) | 0.644 | (0.533, 0.778) | <0.001 |
| **Race, N (%)** |  |  |  |  |
| **White** | 12940 (71.09%) | Ref. |  |  |
| **Asian** | 529 (2.91%) | 0.762 | (0.404, 1.439) | 0.403 |
| **African American** | 1228 (6.75%) | 0.889 | (0.598, 1.323) | 0.563 |
| **Unknown race** | 3505 (19.26%) | 1.150 | (0.915, 1.445) | 0.231 |
| **BMI** | 28.71 ± 6.61 | 0.988 | (0.973, 1.003) | 0.104 |
| **Age range, N (%)** |  |  |  |  |
| **18-40** | 2946 (16.18%) | Ref. |  |  |
| **41-60** | 7554 (41.50%) | 2.783 | (1.739, 4.453) | <0.001 |
| **61-80** | 7098 (39.0%) | 5.054 | (3.195, 7.992) | <0.001 |
| **> 81** | 604 (3.32%) | 15.245 | (9.087, 25.578) | <0.001 |
| **Diabetes, N (%)** |  |  |  |  |
| **No** | 16060 (88.23%) | Ref. |  |  |
| **Yes (Noninsulin-dependent)** | 1351 (7.42%) | 1.520 | (1.113, 2.076) | 0.009 |
| **Yes (Insulin-dependent)** | 791 (4.35%) | 2.601 | (1.892, 3.576) | <0.001 |
| **Smoking status, N (%)** |  |  |  |  |
| **No** | 14676 (80.63%) | Ref. |  |  |
| **Yes** | 3526 (19.37%) | 1.131 | (0.901, 1.420) | 0.287 |
| **Severe COPD, N (%)** |  |  |  |  |
| **No** | 17380 (95.48%) | Ref. |  |  |
| **Yes** | 822 (4.52%) | 2.523 | (1.850, 3.441) | <0.001 |
| **CHF, N (%)** |  |  |  |  |
| **No** | 18143 (99.68%) | Ref. |  |  |
| **Yes** | 59 (0.32%) | 7.142 | (3.491, 14.613) | <0.001 |
| **Hypertension, N (%)** |  |  |  |  |
| **No** | 11235 (61.72%) | Ref. |  |  |
| **Yes** | 6967 (38.28%) | 2.277 | (1.886, 2.750) | <0.001 |
| **Dialysis** |  |  |  |  |
| **No** | 18146 (99.69%) | Ref. |  |  |
| **Yes** | 56 (0.31%) | 6.599 | (3.104, 14.031) | <0.001 |
| **Disseminated cancer, N (%)** |  |  |  |  |
| **No** | 14228 (78.17%) | Ref. |  |  |
| **Yes** | 3974 (21.83%) | 2.877 | (2.382, 3.475) | <0.001 |
| **Steroid use for a chronic condition N (%)** |  |  |  |  |
| **No** | 15459 (84.93%) | Ref. |  |  |
| **Yes** | 2743 (15.07%) | 2.343 | (1.904, 2.882) | <0.001 |
| **Bleeding disorders N (%)** |  |  |  |  |
| **No** | 17829 (97.95%) | Ref. |  |  |
| **Yes** | 373 (2.05%) | 2.266 | (1.430, 3.590) | <0.001 |
| **Preoperative transfusions N (%)** |  |  |  |  |
| **No** | 18139 (99.65%) | Ref. |  |  |
| **Yes** | 63 (0.35%) | 5.757 | (2.726, 12.158) | <0.001 |
| **Preoperative systemic sepsis N (%)** |  |  |  |  |
| **No** | 17531 (96.31%) | Ref. |  |  |
| **SIRS** | 660 (3.63%) | 2.597 | (1.852, 3.640) | <0.001 |
| **Septic Shock** | 11 (0.06%) | 9.188 | (1.979, 42.657) | 0.005 |
| **Serum Na** | 138.63 ± 3.18 | 0.911 | (0.887, 0.936) | <0.001 |
| **BUN** | 17.33 ± 8.07 | 1.043 | (1.036, 1.050) | <0.001 |
| **Cr** | 0.86 ± 0.45 | 1.244 | (1.116, 1.386) | <0.001 |
| **WBC** | 9.5 ± 4.46 | 1.073 | (1.056, 1.090) | <0.001 |
| **PLT** | 244.15 ± 76.45 | 0.998 | (0.997, 0.999) | 0.001 |
| **HCT** | 40.32 ± 4.81 | 0.924 | (0.908, 0.939) | <0.001 |

**Supplementary table 2 Relationship between preoperative HCT and 30-day postoperative mortality in the three binary logistic regression models**

| **Exposure** | **Model 1 (OR, 95% CI, P)** | **Model 2 (OR, 95% CI, P)** | **Model 3 (OR, 95% CI, P)** |
| --- | --- | --- | --- |
| **HCT** | 0.919 (0.903, 0.935) <0.001 | 0.915 (0.899, 0.932) <0.001 | 0.940 (0.922, 0.958) <0.001 |

Model 1 (nonadjusted model): not adjusted for any covariates

Model 2 (minimally adjusted model): adjusted for sex, race, and age ranges

Model 3 (fully adjusted model): adjusted for sex, race, BMI, age range, CHF, diabetes, smoking status, severe COPD, dialysis, hypertension, steroid use for chronic conditions, disseminated cancer, bleeding disorders, preoperative transfusions, preoperative systemic sepsis, serum Na, BUN, Cr, WBC, and PLT

Ref: reference; OR, odds ratios; CI: confidence interval
